# Periodontitis aggravates kidney injury by upregulating STAT1 expression in a mouse model of hypertension

**DOI:** 10.1002/2211-5463.13081

**Published:** 2021-02-19

**Authors:** Qin Yang, Handong Ding, Wei Wei, Jie Liu, Jiajia Wang, Jie Ren, Weicheng Chan, Min Wang, Liang Hao, Jinle Li, Yuan Yue

**Affiliations:** ^1^ State Key Laboratory of Oral Diseases National Clinical Research Center for Oral Diseases Department of Prosthodontics West China Hospital of Stomatology Sichuan University China; ^2^ Ningbo Stomatological Hospital China

**Keywords:** chronic kidney disease, hypertension, periodontitis, STAT1

## Abstract

Periodontitis is an autoimmune disease of periodontal tissues initiated by plaque. It is known that there is a close connection between periodontitis and CKD with hypertension, but the underlying mechanisms are unknown. STAT1 has been reported to play a regulatory role in hypertension and chronic kidney disease (CKD). Here, we investigated whether STAT1 regulates periodontitis‐mediated aggravation of kidney injury with accompanying hypertension. A hypertensive renal injury mouse model was established with *Nos3* knockout mice, and a periodontitis model was established by implantation with the oral bacteria *Porphyromonas gingivalis*. The mice were intraperitoneally injected with a STAT1 inhibitor. Periodontitis aggravated kidney injury in hypertensive mice, and upregulation of STAT1 was observed when both periodontitis and hypertension were present; furthermore, STAT1 inhibitor moderated this effect. Moreover, we observed that periodontitis promoted the upregulation of inflammatory and fibrosis gene expression in the kidneys of hypertensive mice. In addition, STAT1 inhibition decreased the expression of pro‐inflammatory and pro‐fibrotic cytokines in the kidney lesion area. Periodontitis augmented the expression of inflammatory and fibrosis genes by upregulating the expression of STAT1, thereby aggravating kidney injury in the hypertensive mouse model.

## Graphical abstract

Here, we report that STAT1 expression is augmented in a mouse model of periodontitis with hypertension, and this results in upregulation of inflammatory and fibrosis genes, which aggravate hypertensive renal injuries. Injection of fludarabine alleviates kidney injury in this model. Our findings suggest a new target for the treatment of periodontitis in patients with CKD.

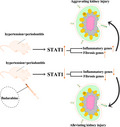

**Abbreviations**
ABC: alveolar crest
CEJ: cemento‐enamel junction
CKD: chronic kidney disease
CT: computed tomography
GAPDH: glyceraldehyde 3‐phosphate dehydrogenase
HRP: horseradish peroxidase
IFN: interferon
IHC: immunohistochemistry
IL: interleukin
NO: nitric oxide
PAS: Periodic acid–Schiff
qRT–PCR: quantitative reverse transcription–polymerase chain reaction
STAT: signal transducer and activator of transcription
TGF: tumor growth factor
TLRs: Toll‐like receptors
TNF: tumor necrosis factor

## Introduction

Periodontitis is considered an autoimmune disease of periodontal tissues initiated by plaque, which can form periodontal pockets, cause alveolar bone resorption, tooth loosening, and eventual tooth loss [1]. In addition to impacting masticatory function and aesthetics, periodontitis can affect systematic conditions and cause systemic diseases, such as hypertension, cardiovascular diseases, and diabetes [2, 3]. Periodontitis may affect the development of hypertension; systolic and diastolic blood pressures of hypertension patients with periodontitis were higher than those of patients without periodontitis, and blood pressure and serum inflammatory factor levels were decreased in patients with both periodontitis and hypertension following periodontal treatment [4, 5]. A previous study also indicated that periodontitis may augment the immune response against disease in subjects with hypertension [6].

Chronic kidney disease (CKD) is characterized by renal structural and functional disorders due to various causes. Its pathological features include arteriosclerosis, glomerulosclerosis, and interstitial fibrosis [7]. Recently, numerous studies have identified a close link between periodontitis and CKD. Fish *et al*. determined that adults with periodontitis were twice as likely to develop CKD compared to that in adults without periodontitis, indicating that periodontitis is a risk factor for CKD [8]. In addition, a systematic review by Chambrone *et al*. demonstrated that the periodontitis treatment could ameliorate the glomerular filtration rate in patients with CKD [9]. Furthermore, Kshirsagar *et al*. found high levels of serum immunoglobulin G antibodies against periodontal pathogens in patients with CKD [10]. Moreover, CKD dialysis patients had a higher prevalence rate of severe periodontitis and deeper periodontal pockets than those without CKD [11]. Furthermore, previous studies have shown that hypertension is one of the main causes of CKD. Hypertensive nephropathy is defined as hypertension‐related nondiabetic CKD with or without moderate proteinuria, and its pathological features include arteriosclerosis, glomerulosclerosis, and interstitial fibrosis [4]. Notably, periodontitis shows a prevailing trend in hypertensive patients [12]. However, whether periodontitis can cause more severe tissue degeneration in patients with CKD and hypertension remains unknown.

The transcription factor signal transducer and activator of transcription (STAT) can directly transfer signals from cell membrane receptors to the nucleus, without the assistance of secondary messengers, and participate in gene expression regulation [13]. STAT is widely involved in cell proliferation, apoptosis, and tumorigenesis, and is also related to the occurrence and development of a variety of diseases [13]. Activation of the JAK/STAT1 pathway can upregulate the expression of pro‐inflammatory factor interleukin (IL)‐1 and pro‐fibrotic factor tumor growth factor (TGF)‐β in the kidney; thus, STAT1 may be a potential therapeutic target for CKD [14]. Saraiva *et al*. revealed the association between a STAT1 gene polymorphism and the development of severe periodontitis, while Haftcheshmeh *et al*. showed that STAT1 is upregulated in the periodontal tissue of patients with periodontitis [15, 16]. Expression of STAT1 was also upregulated in periodontal tissues during the early development stage of experimental periodontitis in rats [17]. Additionally, STAT1 has been shown to mediate hypertension associated with periodontitis bone resorption [18]. However, the specific mechanism underlying STAT1 expression in periodontitis and hypertensive renal injury has not been reported.

In this study, an experimental periodontitis model was established in hypertensive mice (*Nos3^−/−^*) to investigate the effect of periodontitis on hypertension‐related kidney damage. The mechanism responsible for STAT1 expression in periodontitis and hypertension‐related renal injury was explored by detecting changes in STAT1 levels and the expression of inflammation and fibrosis‐related genes in mouse kidney tissues.

## Materials and methods

### Animals

Animal experiments were approved by the Institutional Animal Care and Use Committee of Sichuan University (WCCSIRB‐D‐2015‐030). Four‐week‐old, female, *Nos3^−/−^* and *Nos3^+/+^* mice were obtained from the Model Animal Research Center of Nanjing University. Homozygous *Nos3^−/−^* mice lack NO synthase, leading to a hypertensive phenotype [19]. After acclimation for four weeks, mice were divided into the following eight groups, with 10 mice per group: *Nos3^+/+^* mice without or with STAT1 inhibitor fludarabine injection (C and CI, respectively); *Nos3^−/−^* mice without or with STAT1 inhibitor fludarabine injection (H and HI, respectively); bacteria‐implanted *Nos3^+/+^* mice without or with STAT1 inhibitor fludarabine injection (P and PI, respectively); and bacteria‐implanted *Nos3^−/−^* mice without or with STAT1 inhibitor fludarabine injection (PH and PHI, respectively). Mice were fed standard food and water at the State Key Laboratory for Oral Disease. The experiment was repeated independently in triplicate.

### Bacterial infection

The density of *Porphyromonas gingivalis W50* (ATCC: 53978) bacteria was controlled at 10^9^–10^10^ CFU·mL^−1^, and 3% carboxymethyl cellulose solution was added by half‐volume to increase bacterial viscosity. Following this, a small dental swab dipped in the bacterial mixture was used to brush the oral cavity of the mice for eight consecutive days to induce periodontitis.

### Administration of the STAT1 inhibitor

After implanting with bacteria, the mice in the inhibitor groups were intraperitoneally injected with 300 μL (10 mg·mL^−1^) fludarabine solution (CAT#F2773; Sigma‐Aldrich, St. Louise, MO, USA) [20], a specific inhibitor of STAT1, and the mice in the noninhibitor groups were intraperitoneally injected with the same volume of 1 × PBS solution. The injection was administered for 4 weeks, at three times a week.

### Tissue harvesting and preparation of samples

Mice were euthanized by anesthetic overdose 56 days after administration of the bacterial mixture, and the kidneys were surgically removed. The right kidney was cut in half along the long axis: Half was stored at −80 ºC for RNA extraction, and the other half was stored at −80 °C for protein extraction. The left kidney was immersed in 4% paraformaldehyde for 24 h, soaked in tap water for 8 h, soaked in 50% alcohol solution for 30 min, and finally stored in 70% alcohol solution at 4 °C for future histopathological study. Mouse maxilla was collected and treated as the left kidney prior to the computed topography (CT) analysis.

### Microcomputed tomography scanning and analysis

Mouse maxilla was scanned by micro‐CT (Scanco Medical, Wangen‐Brüttisellen, Switzerland). The scanning conditions were set as follows: current, 145 mA; voltage, 55 kVp; resolution, 12 μm; and integration time, 300 ms. After scanning, CT data were reconstructed using MIMICS software (Materialise, Leuven, Belgium) and the distance from the alveolar crest (ABC) to the cemento‐enamel junction (CEJ) between the first and second molars was measured.

### Periodic acid‐Schiff staining

Periodic acid–Schiff (PAS) staining was conducted according to the manufacturer's instructions (Solarbio, Beijing, China), and the sections were examined under the microscope (Leica, Wetzlar, Germany). The glomerular and PAS‐positive areas in the glomeruli were measured using imagej software (version 1.52) [21], and the ratio between the two was calculated.

### Masson staining

Masson staining was conducted following the manufacturer's instructions (Solarbio, Beijing, China) and examined under the microscope (Leica). The area of blue collagen fibers was measured using imagej software (version 1.52), and its percentage of the whole visual field was calculated.

### Immunohistochemistry (IHC)

The expression and localization of STAT1 proteins in the kidney were detected by anti‐STAT1 (Cell Signaling Technology Inc., Danvers, MA, USA) antibody staining. The area of STAT1‐positive cells was analyzed by quantitative morphometry using imagej software (version 1.52).

### Protein exaction and western blot

Total protein was extracted from kidney tissue samples using a total protein extraction kit (Signalway Antibody; College Park, MD, USA). Proteins were separated using SDS/PAGE and then transferred to membranes. Western blotting was performed using the following primary antibodies: anti‐STAT1 (Cell Signaling Technology Inc.) and anti‐GAPDH (Cell Signaling Technology Inc.). Next, the membrane was incubated with HRP‐conjugated antibodies (Signalway Antibody). The ChemiDoc™ MP Imaging System (Bio‐Rad Laboratories, Hercules, CA, USA) was used.

### ***RNA extraction and qRT*** *–* ***PCR***

Total RNA was extracted from kidney tissue samples according to the instructions provided with the NucleoTrap^®^ mRNA Midi Kit (Takara Bio Inc., Kusatsu, Japan). The RevertAid First‐Strand cDNA Synthesis Kit (Thermo Fisher Scientific, Waltham, MA, USA) was used to perform the reverse transcription of the total RNA. Following this, SYBR^®^ Green PCR Master Mix (Sigma‐Aldrich) was used for qRT–PCR, and mRNA expression of the selected genes was analyzed using primers (5′–3′) listed in Table S1. The fold change was calculated using the ΔΔCt method.

### Statistical analysis and data quantification

Data are presented as the mean ± standard deviation (SD) of the independent samples. Data were analyzed by two‐tailed Student′s *t*‐test and one‐way analysis of variance (ANOVA). The Mann–Whitney *U*‐test was applied for nonparametric data. Values of *P* < 0.05 or *U* > 1.96 were considered statistically significant.

## Results

### Periodontitis model was successfully constructed by oral bacterial implantation

**Fig. 1:**
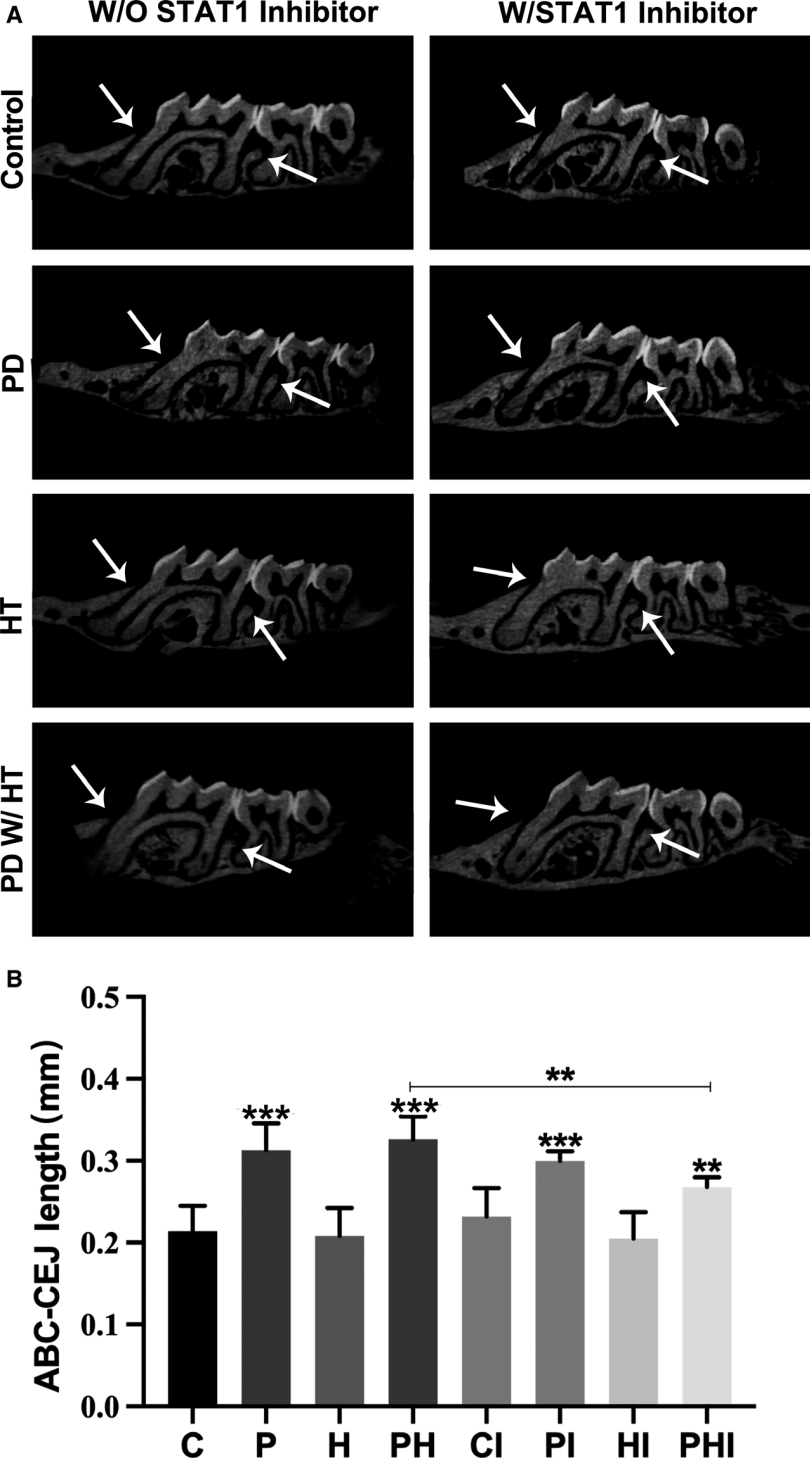
The periodontitis model was successfully constructed by oral bacterial implantation. (A) Microcomputer topography of the maxilla along the long axis. White arrows indicate the location of the alveolar crest. PD, periodontitis; HT, with hypertension; W/, with; W/O, without; STAT1 inhibitor, injected with STAT1 inhibitor. (B) Quantitative analysis of the distance from the alveolar crest to the cemento‐enamel junction between the first and second molars. C, control group; CI, control + inhibitor group; P, periodontitis group; PI, periodontitis + inhibitor group; H, hypertension group; HI, hypertension + inhibitor group; PH, periodontitis + hypertension group; PHI, periodontitis + hypertension + inhibitor group. Data are presented as the mean ± SD (n = 10 per group) of independent samples, and experiments were repeated three times. Differences between two groups were compared using one‐way analysis of variance (ANOVA). *p < 0.05, **p < 0.01, and ***p < 0.001. The asterisk (with no line connection) on the column of this group represents the statistical difference between it and the control group.

To verify the success of bacterial implantation and development of periodontitis, tooth samples from each group were analyzed using micro‐CT (Fig. 1A). The results show the resorption of alveolar bone of each group. By observing the between‐group differences in alveolar crest position, we found that substantially more alveolar bone resorption occurred in groups implanted with bacteria (P, PI, PH, and PHI) compared to those not implanted with bacteria (C, CI, H, and HI). The distance from ABC to CEJ between the first and second molars was larger in the P, PI, PH, and PHI groups than that in the group C; however, there was no significant difference among the C, CI, H, and HI groups (Fig. 1B). The groups implanted with bacteria showed considerable alveolar bone resorption, indicating that the experimental induction of periodontitis was successful. However, when compared with the noninhibitor group, we found that both the distance from ABC to CEJ and bone resorption area decreased in the inhibitor group, with the distance from ABC to CEJ having the most obvious decline when periodontitis and hypertension coexisted, indicating that administration of STAT1 inhibitor could ameliorate the bone resorption.

### Periodontitis aggravates glomerular mesangial dilation and tubulointerstitial fibrosis in hypertensive mouse model

**Fig. 2:**
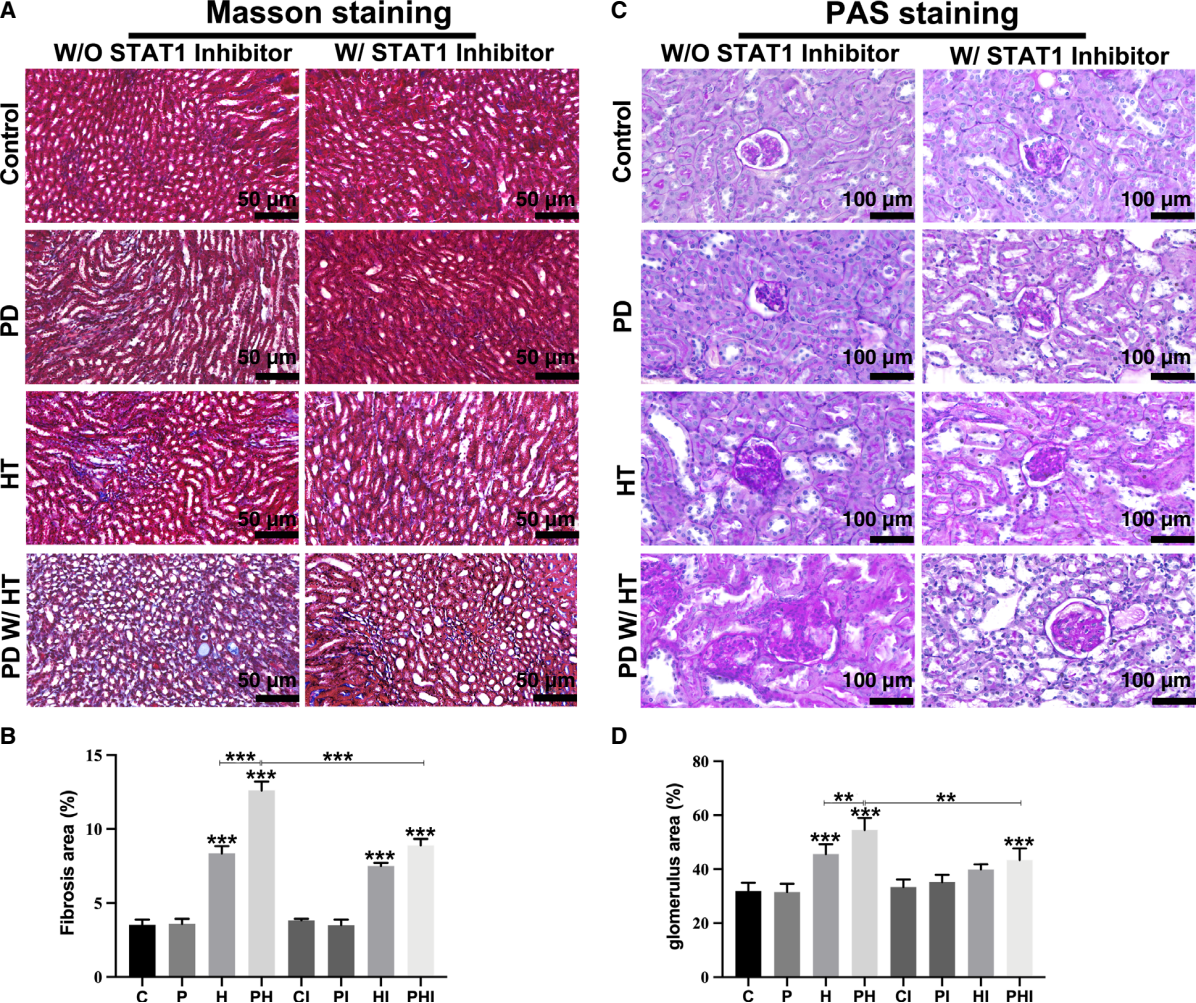
Periodontitis aggravates glomerular mesangial dilation and tubulointerstitial fibrosis in the hypertensive mouse model. (A) Periodic acid–Schiff (PAS) staining of glomeruli. Scale bar: 50 µm. (B) Semi‐quantitative analysis of the ratio of glomerular mesangial matrix to glomerular area. (C) Masson staining of kidney tissues. Scale bar: 100 µm. (D) Proportion of renal tubulointerstitial fibrosis area. C, control group; CI, control + inhibitor group; P, periodontitis group; PI, periodontitis + inhibitor group; H, hypertension group; HI, hypertension + inhibitor group; PH, periodontitis + hypertension group; PHI, periodontitis + hypertension + inhibitor group. Data are presented as the mean ± SD (*n* = 10 per group) of independent samples, and experiments were repeated three times. Differences between two groups were compared using one‐way analysis of variance (ANOVA). *P < 0.05, **P < 0.01, and ***P < 0.001. The asterisk (with no line connection) on the column of this group represents the statistical difference between it and the control group.

The blood pressure of mice in each group is shown in Figure S1, and the diastolic and systolic blood pressures of the hypertension groups (H, HI, PH, and PHI) were higher than those of the control groups (C and CI). To determine the effect of periodontitis on renal injury in hypertensive mice, PAS staining was used to detect glomerular mesangial dilation and Masson staining was used to detect renal tubulointerstitial fibrosis. Following PAS staining, a large number of dark blue nuclei were observed and the mesangial matrix outside the nucleus was stained purplish red, exhibiting PAS‐positive staining. In the groups without hypertension (C, CI, P, and PI), the mesangial matrix in the glomeruli was loosely arranged, accounting for only a small part of the glomerulus (Fig. 2A). While the mesangial matrix significantly increased in the hypertension groups H and HI (Fig. 2A), the groups with both hypertension and periodontitis (PH and PHI) had the most severe mesangial matrix dilation, which almost filled the whole glomerulus (Fig. 2A). The mesangial matrix of the PHI group was lower than that of the PH group (Fig. 2A). The ratio of glomerular mesangial matrix to glomerular area was semi‐quantitatively analyzed, revealing that the glomerular ratios in the H, HI, PH, and PHI groups were significantly higher than that in the control group (*p* < 0.05) (Fig. 2B). The glomerular ratio in the PH group was significantly higher than that in the H group (*p* < 0.01), and the glomerular ratio of the PHI group was lower than that in the PH group (Fig. 2B). Masson staining showed the area of fibrosis by staining blue collagen fibers between the renal tubules. In the groups of mice without hypertension, only a few collagen fibers were observed between the renal tubules, while the area of fibrosis was significantly increased in the hypertension groups H and HI. The fibrotic area in the PH group was the largest, almost filling the whole field of view, while the fibrotic area in the PHI group, which was treated with an inhibitor, was smaller than that in the PH group (Fig. 2C). The ratio of fibrotic area to the whole field of view was semi‐quantitatively analyzed, demonstrating that the fibrotic area ratio in hypertensive mice was significantly higher (*P* < 0.05) than that of mice in the control groups. The fibrotic area ratio in the PH group was higher than that in the H group, while the ratio in the PHI group was significantly lower (*P* < 0.01) than that in the PH group (Fig. 2D). The aforementioned results indicate that the mesangial matrix dilation and renal tubulointerstitial fibrosis in hypertensive mice were more significant than those in normal mice, and periodontitis might further aggravate these changes. Treatment with the STAT1 inhibitor alleviated the pathophysiological changes associated with periodontitis in the hypertension groups, but had no significant effect on the other groups. Additionally, the physiological data (kidney weight and body weight, urea nitrogen, serum creatinine) of mice in each group could also support the conclusion above (Figs S2, 3).

### Periodontitis and hypertension upregulate expression of STAT1 protein in the kidney

**Fig. 3:**
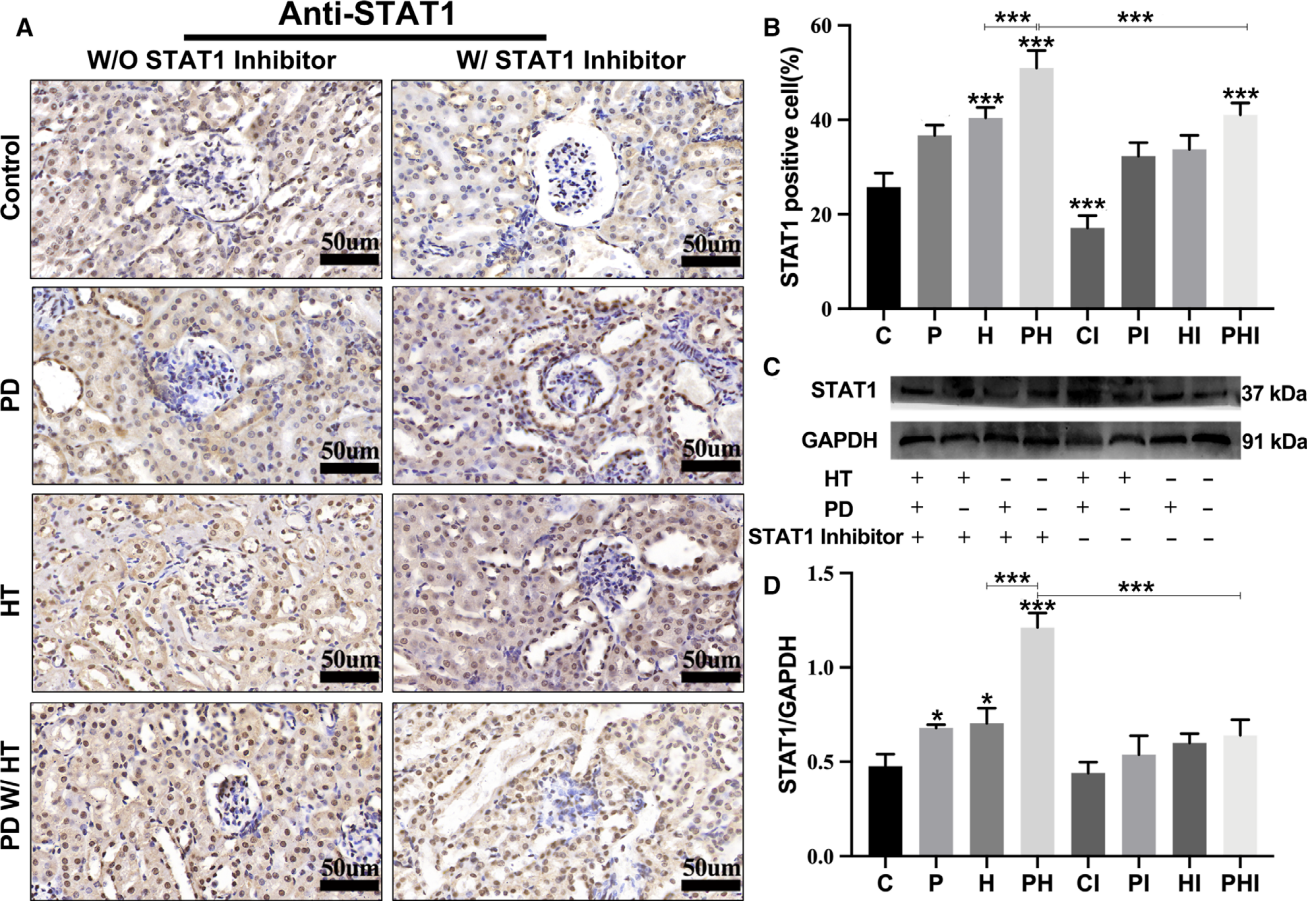
Periodontitis and hypertension upregulate the expression of STAT1 protein in mouse kidneys. (A) Immunohistochemistry of STAT1. Scale bar: 50 um. (B) Semi‐quantitative analysis of the proportion of STAT1‐positive cells in the kidneys. (C) Western blot of STAT1 protein expression, with GAPDH as the internal reference protein. (D) Relative expression of STAT1 protein in the kidneys. C, control group; CI, control + inhibitor group; P, periodontitis group; PI, periodontitis + inhibitor group; H, hypertension group; HI, hypertension + inhibitor group; PH, periodontitis + hypertension group; PHI, periodontitis + hypertension + inhibitor group. Data are presented as the mean ± SD (*n* = 10 per group) of independent samples, and experiments were repeated three times. Differences between two groups were compared using one‐way analysis of variance (ANOVA). *P < 0.05, **P < 0.01, and ***P < 0.001. The asterisk (with no line connection) on the column of this group represents the statistical difference between it and the control group

STAT1 is a cytoplasmic protein that appears as yellowish‐brown STAT1‐positive cells during IHC anti‐STAT1 antibody staining. A certain proportion of STAT1‐positive cells remained in the control group (Fig. 3A). IHC and western blot results revealed that the abundance of STAT1‐positive cells increased in the presence of periodontitis or hypertension. The proportion of STAT1‐positive cells was highest when hypertension and periodontitis coexisted (PH), and the proportion decreased significantly in the inhibitor group (PHI) (Fig. 3A,B). Further, the expression of STAT1 was highest in the PH group and STAT1 protein expression in this group decreased most significantly (*P* < 0.05) after treatment with the STAT1 inhibitor (Fig. 3C,D). Additionally, the expression of P‐STAT1 was consistent with the data above, which markedly increased in the periodontitis group and the hypertension group, and was the highest in the periodontitis with hypertension group, indicating that periodontitis and hypertension could promote the activation of STAT1, while inhibition of STAT1, following injection of fludarabine, prevented the activation of STAT1 (Fig. S5).

### STAT1 affects expression of inflammatory and fibrosis genes during periodontitis and aggravates renal injury in hypertensive mice

**Fig. 4:**
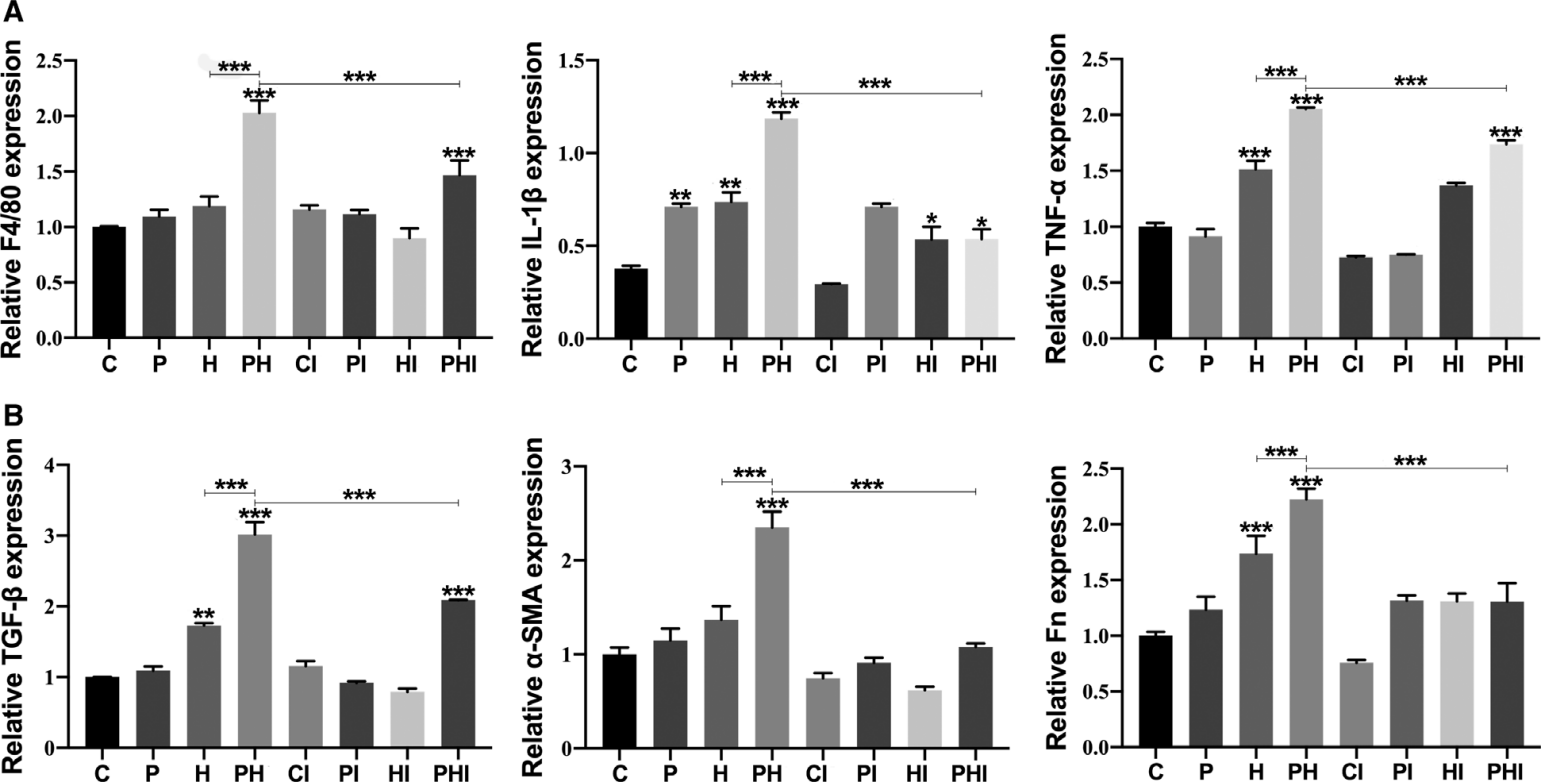
STAT1 affects the expression of inflammatory and fibrosis genes and aggravates renal injury in hypertensive mice with periodontitis. (A) Relative expression levels of *F4/80*, *Tnf‐α*, and *Il‐1β*. (B) Relative expression levels of *TGF‐β*, *α‐SMA*, and fibronectin. C, control group; CI, control + inhibitor group; P, periodontitis group; PI, periodontitis + inhibitor group; H, hypertension group; HI, hypertension + inhibitor group; PH, periodontitis + hypertension group; PHI, periodontitis + hypertension + inhibitor group. Data are presented as the mean ± SD (*n* = 10 per group) of independent samples, and experiments were repeated three times. Differences between two groups were compared using one‐way analysis of variance (ANOVA). *P < 0.05, **P < 0.01, and ***P < 0.001. The asterisk (with no line connection) on the column of this group represents the statistical difference between it and the control group.

Through the detection of inflammatory and fibrosis genes in mouse renal tissue samples from each group, we found that the expression of inflammation‐related genes *F4/80*, *Tnf‐α*, and *Il‐1β* increased compared with those in the control group, with the most significant increase observed in the PH group (*P* < 0.0001). The expression of inflammation‐related genes in the corresponding groups decreased after treatment with the STAT1 inhibitor (Fig. 4A). Besides, *STAT3* was significantly upregulated in the periodontitis group, while No3 knockout reduced its expression in the hypertension group and the periodontitis with hypertension group. Following inhibition of STAT1, the expression of STAT3 increased in the inhibitor group, suggesting that STAT1 could inhibit the anti‐inflammatory function of STAT3 (Fig. S4). Similar to the changes in expression of inflammation‐related genes, the expression of fibrosis‐related genes *TGF‐β*, *α‐SMA*, and fibronectin also decreased after treatment with the STAT1 inhibitor (Fig. 4B). The aforementioned results suggest that inhibiting the expression of STAT1 had a diminishing effect on the expression of both inflammatory and fibrosis genes.

## Discussion

Several studies have examined the effects of periodontitis on CKD [8, 9, 10, 11]. Czesnikiewicz‐Guzik *et al*. found that severe periodontitis was related to poor renal conditions in predialytic CKD patients [22]. Chopra *et al*. suggested that the molecular mechanism responsible for periodontitis aggravating CKD might be explained by periodontal pathogenic products, such as lipopolysaccharides, which activate Toll‐like receptors (TLRs) to produce pro‐inflammatory cytokines (IL‐6 and TNF‐α) that aggravate kidney tissue damage [23].

Previous research has shown a strong relationship between CKD and hypertension. A cohort study found that apparent treatment‐resistant hypertension was associated with high risk of detrimental results in CKD patients [24]. Decreasing systemic blood pressure and intraglomerular pressure has led to the development of novel therapeutic strategies for patients with renal insufficiency [7]. Additionally, this may benefit blood pressure control in CKD patients by targeting specific inflammatory pathways [25].

However, few studies have elucidated whether periodontitis affects kidney injury when accompanied by hypertension. In our study, we successfully established a hypertensive renal injury model with periodontitis. PAS and Masson staining results revealed that periodontitis aggravated glomerular mesangial dilation and tubulointerstitial fibrosis in the hypertensive mouse model, indicating that periodontitis aggravates hypertensive kidney damage.

STAT1 has been proven to regulate inflammation [26]. Studies have shown that patients with invasive and chronic periodontitis express higher STAT1 levels than those in healthy individuals [16]. Zhou *et al*. found that JAK2/STAT1 was significantly enhanced during the aging of human mesangial cells induced by AngII [27]. Frank et al found that fludarabine was a specific STAT1 inhibitor, which could inhibit the cytokine‐induced activation of STAT1 and STAT1‐dependent gene transcription, and it also caused a specific depletion of STAT1 protein (and mRNA) but not of other STATs [28]. Thus, we administered fludarabine to investigate the role of STAT1 in the aggravation of periodontitis‐induced renal damage in hypertensive mice. While STAT1 expression was completely inhibited in all groups, it was dramatically decreased in hypertensive mice with periodontitis. These data suggested that STAT1 plays a crucial role in the concomitance of the two diseases.

Inflammation and fibrosis play a significant role in the occurrence and development of hypertensive nephropathy. Our results indicated that the superposition of periodontitis and hypertension significantly upregulated *IL‐1 β, TNF‐α,* and *F4/80*, which is consistent with the pathological manifestations of glomerular mesangial dilation and tubulointerstitial fibrosis. We hypothesize that STAT1 may aggravate renal damage in hypertensive mice by upregulating inflammatory genes. Other studies on the STAT pathway have shown a relationship between STAT1 and inflammation. Sikorski *et al*. found that after IFN‐γ binds to the corresponding membrane receptor, the activation of the receptor‐coupled JAK kinase results in the phosphorylation of STAT1, and the activated STAT1 forms a dimer directly into the nucleus, thus starting the transcription of many cytokines, such as IRF8, IP‐10, and CCL5. IRF8 can then promote the activation of the downstream TLR pathway and produce various pro‐inflammatory factors such as TNF‐α and IL‐1β [29]. Wei *et al*. found that inhibiting STAT1 decreased macrophage infiltration and the production of inflammatory cytokines [18].

Similar to the inflammatory genes, hypertension can upregulate *TGF‐β, Fn,* and *α‐SMA*; however, periodontitis alone had no significant effect on the expression of the three fibrosis‐related genes. Nevertheless, the three genes were significantly upregulated when periodontitis and hypertension coexisted, and significantly downregulated after inhibiting the expression of STAT1. Therefore, we speculate that STAT1 may aggravate renal damage in hypertensive mice by upregulating fibrosis genes. Ying *et al*. also found that ROS (reactive oxygen species) can activate STAT1 in immunoglobulin‐related renal injury in animal experiments. However, the specific role of STAT1 in the signal transduction of TGF‐β is not clear [30].

In conclusion, our research demonstrated that STAT1 expression was augmented by periodontitis, resulting in the upregulation of inflammatory and fibrosis genes, which aggravated the hypertensive renal injuries in a mouse model. Our findings suggest a new target for the treatment of periodontitis in patients with CKD.

## Author contributions

QY and YY conceived and designed the project; QY, H‐DD, JL, and WW acquired the data; J‐JW, W‐CZ, HL, MW, J‐LL, and JR analyzed and interpreted the data; and QY wrote the manuscript.

## Conflict of Interest

The authors declare no conflict of interest.

## Supporting information


**Fig S1.** Blood pressures of each group.

**Fig S2.** Body weight, as well as kidney appearance and weight of each group.

**Fig S3.** (A) Serum creatinine levels of each group. (B) Urea nitrogen levels of each group. C, control group; CI, control + inhibitor group; P, periodontitis group; PI, periodontitis + inhibitor group; H, hypertension group; HI, hypertension + inhibitor group; PH, periodontitis + hypertension group; PHI, periodontitis + hypertension + inhibitor group. Data are presented as the mean ± SD (n=10 per group) of independent samples and experiments were repeated three times. Differences between two groups were compared using one‐way analysis of variance (ANOVA). *p < 0.05, **p < 0.01, ***p < 0.001. The asterisk (with no line connection) on the column of this group represents the statistical difference between it and the control group.

**Fig S4.** Relative *STAT3* expression in each group.

**Fig S5.** Expression of P‐STAT1 in each group.

**Table S1.** Primers used for qRT‐PCR.

## Data Availability

The datasets used and/or analyzed during the current study are available from the corresponding author upon reasonable request.
