# Supplementary material for: Periodontitis aggravates kidney injury by upregulating STAT1 expression in a mouse model of hypertension
Source: FEBS Open Bio. 2021 Feb 19;11(3):880–9. doi: 10.1002/2211-5463.13081 (PMC7931221; doi:10.1002/2211-5463.13081)
Supplement: Supplementary file 1 — Fig S1. Blood pressures of each group. [file FEB4-11-880-s001.docx]

**
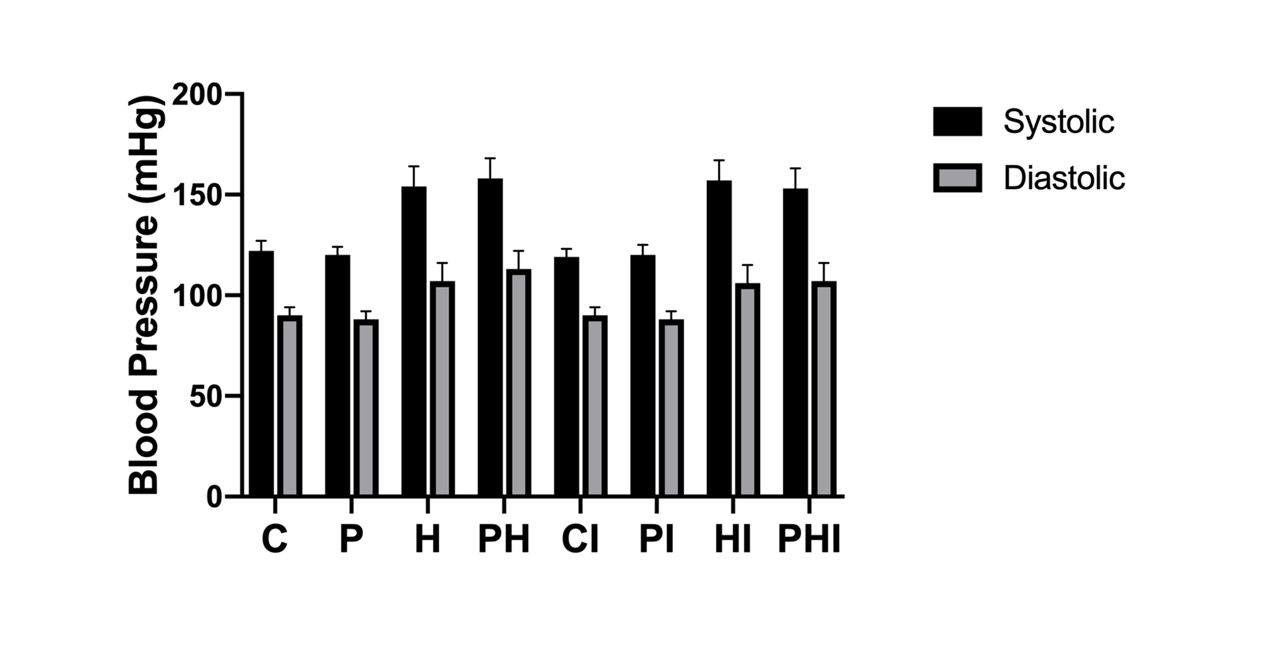
Supplementary Figure 1. Blood pressures of each group.** C, control group; CI, control + inhibitor group; P, periodontitis group; PI, periodontitis + inhibitor group; H, hypertension group; HI, hypertension + inhibitor group; PH, periodontitis + hypertension group; PHI, periodontitis + hypertension + inhibitor group. Data are presented as the mean ± SD of independent samples.
