# Supplementary material for: Periodontitis aggravates kidney injury by upregulating STAT1 expression in a mouse model of hypertension
Source: FEBS Open Bio. 2021 Feb 19;11(3):880–9. doi: 10.1002/2211-5463.13081 (PMC7931221; doi:10.1002/2211-5463.13081)
Supplement: Supplementary file 3 — Fig S3. (A) Serum creatinine levels of each group. (B) Urea nitrogen levels of each group. C, control group; CI, control + inhibitor group; P, periodontitis group; PI, periodontitis + inhibitor group; H, hypertension group; HI, hypertension + inhibitor group; PH, periodontitis + hypertension group; PHI, periodontitis + hypertension + inhibitor group. Data are presented as the mean ± SD (n=10 per group) of independent samples and experiments were repeated three times. Differences between two groups were compared using one‐way analysis of variance (ANOVA). *p < 0.05, **p < 0.01, ***p < 0.001. The asterisk (with no line connection) on the column of this group represents the statistical difference between it and the control group. [file FEB4-11-880-s003.docx]

**
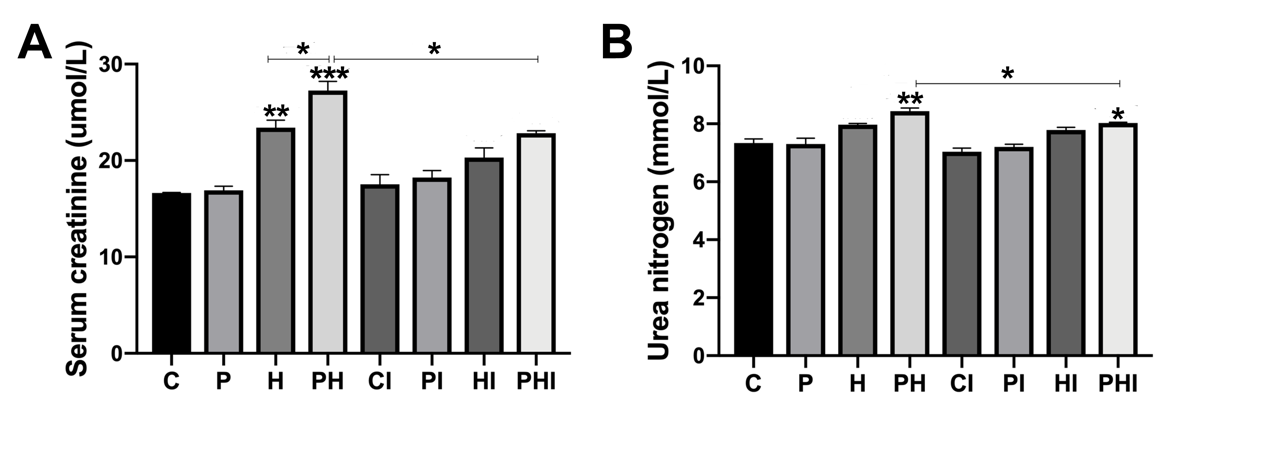
**

**Supplementary Figure 3. (A)** Serum creatinine levels of each group. **(B)** Urea nitrogen levels of each group**.** C, control group; CI, control + inhibitor group; P, periodontitis group; PI, periodontitis + inhibitor group; H, hypertension group; HI, hypertension + inhibitor group; PH, periodontitis + hypertension group; PHI, periodontitis + hypertension + inhibitor group. Data are presented as the mean ± SD of independent samples. **p* < 0.05, ***p* < 0.01, ****p* < 0.001.
