# Supplementary material for: Periodontitis aggravates kidney injury by upregulating STAT1 expression in a mouse model of hypertension
Source: FEBS Open Bio. 2021 Feb 19;11(3):880–9. doi: 10.1002/2211-5463.13081 (PMC7931221; doi:10.1002/2211-5463.13081)
Supplement: Supplementary file 4 — Fig S4. Relative STAT3 expression in each group. [file FEB4-11-880-s004.docx]

**
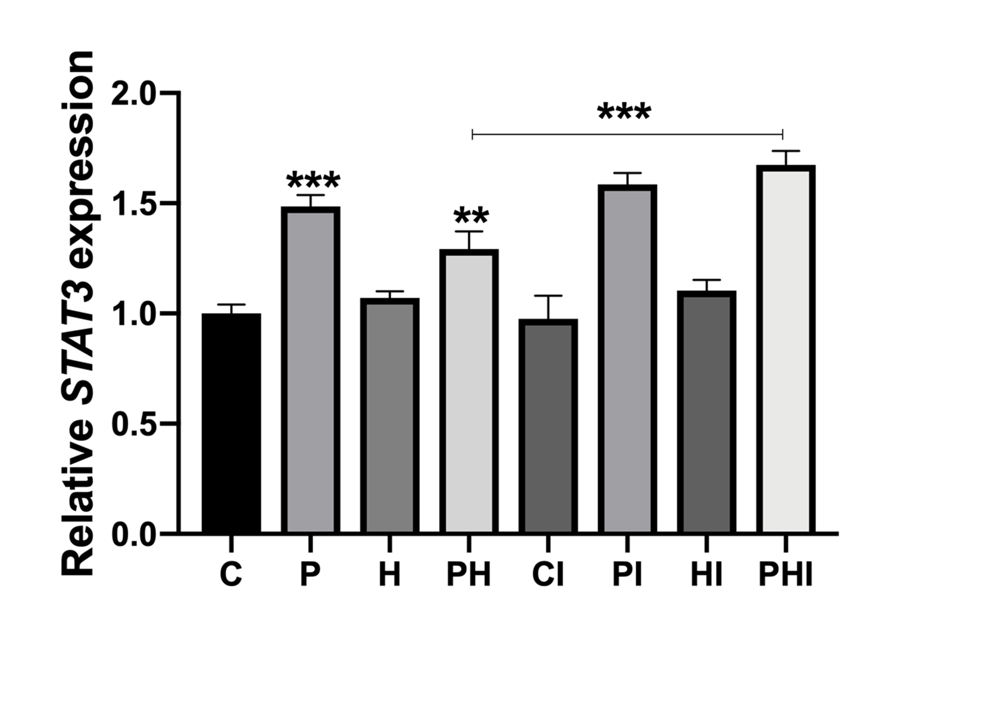
**

**Supplementary Figure 4. Relative *STAT3* expression in each group.** C, control group; CI, control + inhibitor group; P, periodontitis group; PI, periodontitis + inhibitor group; H, hypertension group; HI, hypertension + inhibitor group; PH, periodontitis + hypertension group; PHI, periodontitis + hypertension + inhibitor group. Data are presented as the mean ± SD of independent samples. **p* < 0.05, ***p* < 0.01, ****p* < 0.001.
