# Supplementary material for: Periodontitis aggravates kidney injury by upregulating STAT1 expression in a mouse model of hypertension
Source: FEBS Open Bio. 2021 Feb 19;11(3):880–9. doi: 10.1002/2211-5463.13081 (PMC7931221; doi:10.1002/2211-5463.13081)
Supplement: Supplementary file 6 — Table S1. Primers used for qRT‐PCR. [file FEB4-11-880-s006.docx]

**Supplementary Table 1. Primers used for qRT-PCR**

| **Gene** | **Forward Primer** | **Reverse Primer** |
| --- | --- | --- |
| *IL-1β* | TGCACTACAGGCTCCGAGAT | CGTTGCTTGGTTCTCCTTGT |
| *TNF-α* | AGGGTCTGGGCCATAGAACT | CCACCACGCTCTTCTGTCTAC |
| *F4/80* | TGACAACCAGACGGCTTGTG | GCAGGCGAGGAAAAGATAGTGT |
| *α-SMA* | ACTGGGACGACATGGAAAAG | GTTCAGTGGTGCCTCTGTCA |
| Fibronectin | GGAGTGGCACTGTCAACCTC | ACTGGATGGGGTGGGAAT |
| *TGF-β* | GAGCCCGAAGCGGACTACTA | CACTGCTTCCCGAATGTCTGA |
| *GAPDH* | AGGTTGTCTCCTGCGACTTCA | CCAGGAAATGAGCTTGACAAA |
